# Elucidating Carfilzomib’s Induced Cardiotoxicity in an In Vivo Model of Aging: Prophylactic Potential of Metformin

**DOI:** 10.3390/ijms222010956

**Published:** 2021-10-11

**Authors:** Panagiotis Efentakis, Garyfalia Psarakou, Aimilia Varela, Eleni Dimitra Papanagnou, Michail Chatzistefanou, Panagiota-Efstathia Nikolaou, Costantinos H. Davos, Maria Gavriatopoulou, Ioannis P. Trougakos, Meletios Athanasios Dimopoulos, Ioanna Andreadou, Evangelos Terpos

**Affiliations:** 1 Laboratory of Pharmacology, Faculty of Pharmacy, National and Kapodistrian University of Athens, 15771 Athens, Greece; pefentakis@pharm.uoa.gr (P.E.); pefentakis@yahoo.com (G.P.); michalis.chatzistefanou@gmail.com (M.C.); nayanik@pharm.uoa.gr (P.-E.N.); 2 Cardiovascular Research Laboratory, Clinical, Experimental Surgery & Translational Research Center, Biomedical Research Foundation Academy of Athens, 11527 Athens, Greece; evarela@bioacademy.gr (A.V.); cdavos@bioacademy.gr (C.H.D.); 3 Department of Cell Biology, Biophysics Faculty of Biology, National and Kapodistrian University of Athens, 15771 Athens, Greece; epapanagnou@biol.uoa.gr (E.D.P.); itrougakos@biol.uoa.gr (I.P.T.); 4 Department of Clinical Therapeutics, School of Medicine, National and Kapodistrian University of Athens, 11528 Athens, Greece; mgavria@med.uoa.gr (M.G.); mdimop@med.uoa.gr (M.A.D.); eterpos@med.uoa.gr (E.T.)

**Keywords:** aging, carfilzomib, metformin, cardiotoxicity, Bip, AMPKα

## Abstract

Background: Carfilzomib is a first-line proteasome inhibitor indicated for relapsed/refractory multiple myeloma (MM), with its clinical use being hampered by cardiotoxic phenomena. We have previously established a translational model of carfilzomib cardiotoxicity in young adult mice, in which metformin emerged as a prophylactic therapy. Considering that MM is an elderly disease and that age is an independent risk factor for cardiotoxicity, herein, we sought to validate carfilzomib’s cardiotoxicity in an in vivo model of aging. Methods: Aged mice underwent the translational two- and four-dose protocols without and with metformin. Mice underwent echocardiography and were subsequently sacrificed for molecular analyses in the blood and cardiac tissue. Results: Carfilzomib decreased proteasomal activity both in PBMCs and myocardium in both protocols. Carfilzomib induced mild cardiotoxicity after two doses and more pronounced cardiomyopathy in the four-dose protocol, while metformin maintained cardiac function. Carfilzomib led to an increased Bip expression and decreased AMPKα phosphorylation, while metformin coadministration partially decreased Bip expression and induced AMPKα phosphorylation, leading to enhanced myocardial LC3B-dependent autophagy. Conclusion: Carfilzomib induced cardiotoxicity in aged mice, an effect significantly reversed by metformin. The latter possesses translational importance as it further supports the clinical use of metformin as a potent prophylactic therapy.

## 1. Introduction

Proteasome inhibition has emerged as a pivotal druggable approach in managing multiple myeloma (MM). Proteasome inhibitors such as bortezomib and carfilzomib (Cfz) are currently incorporated in the clinical practice as first-line treatments against MM and relapsed/refractory MM (R/RMM), improving the prognosis of patients and mitigating the disease progression [1,2,3]. Despite the fact that the value of proteasome inhibitors is well recognized, the manifestation of adverse effects hampers their clinical use. The irreversible proteasome inhibitor Cfz is considered a therapy of choice in patients with R/R MM, shown to be preponderant to the reversible proteasome inhibitor bortezomib in terms of event-free survival [2]. However, its clinical application is accompanied by the prevalence of serious cardiovascular side effects, among which acute heart failure (HF) and hypertension are the most frequently observed [4,5].

We have successfully established previously an in vivo translational model of Cfz cardiotoxicity through the application of various dose regimens and time points, and we have concluded that two and four doses of Cfz led to a significant decrease in left ventricular (LV) function, which was orchestrated by an upregulation of protein phosphatase 2A (PP2A) activity leading to decreased AMPKα phosphorylation and downregulation of autophagy in the myocardium [6]. Restoration of AMPKα phosphorylation by the clinically relevant anti-hyperglycemic drug metformin (Met), acting as an AMPKα activator [7], abrogated Cfz-induced cardiomyopathy by restoring myocardial AMPKα-dependent autophagy [6].

MM is considered to be an elderly disease, with its incidence staggering considerably with age [8]. Despite its high frequency, there is limited information regarding the outcome of elderly patients with myeloma. Few clinical studies suggest that advanced age is associated with shorter survival, whereas other studies do not demonstrate such a relationship [9]. Advanced age, functional decline in physical activities, and the presence of comorbidities are associated with a progressive worsening of overall survival, regardless of International Staging System (ISS) stage and MM therapeutic regimen [10]. Considering Cfz’s antitumor potency, it is shown that its efficacy is not compromised by aging, and Cfz-lenalidomide-dexamethasone regimen has a favorable benefit-risk profile for patients with R/R MM, including elderly patients ≥70 years old. However, in Cfz-treated patients, grade ≥3 cardiovascular adverse events, such as hypertension, acute HF, and ischemic heart disease, were presented with higher occurrence among patients ≥70 years old versus patients <70 years old [4].

Age is a major independent determinant of cardiovascular diseases (CVD) [11], with excessive oxidative stress, sustained low-grade inflammation, reduced endoplasmic reticulum stress resistance, genomic instability, and impaired proteostasis being implicated in age-related cardiomyopathy [12]. Notably, HF prevalence increases dramatically in the elderly, as for instance, in the Rochester Epidemiologic Project in Olmsted County, the overall manifestation ratio of HF was elevated with age, reaching 8.4% in those age >75 years old compared with 0.7% in those 45–54 years of age [13]. Regarding that cardiotoxicity phenomena are more evident in carfilzomib therapy in elderly patients and that aging is strongly correlated with CVD manifestation, herein, we sought to investigate the cardiovascular effects of Cfz in an aged murine model and challenge the cardioprotective potential of Met in the aged myocardium.

## 2. Results

### 2.1. Carfilzomib Administration of Two and Four Doses Decreased Proteasome Activity, % Fractional Shortening and Increased Myocardial Oxidative Stress

**Figure 1:**
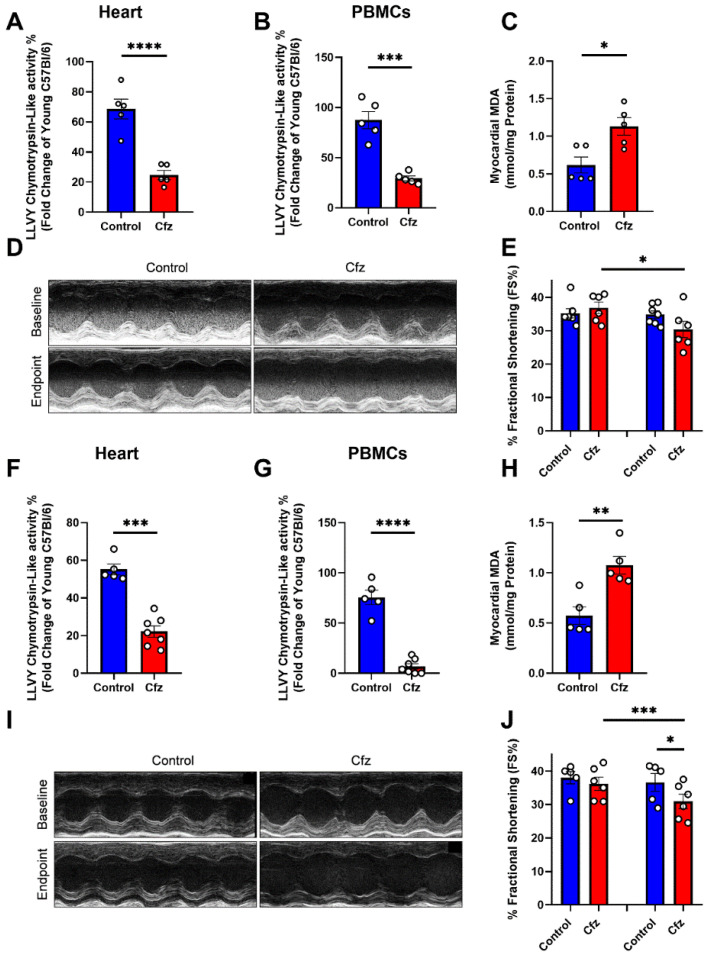
Two and four doses of carfilzomib inhibited proteasomal activity, increased myocardial MDA content, and deteriorated left ventricular function. Graphs of LLVY chemotrypsin-like activity % in the (**A**). heart and (**B**). PBMCs expressed as fold change of young adult (13–14 weeks of age) C57BL/6J mice after two doses of Cfz (*n* = 5 per group). (**C**). Myocardial MDA content (in mmol/mg protein, *n* = 5 per group). (**D**,**E**). Representative M-Mode images and graph of FS% in the two-dose protocol (*n* = 5 per group). Graphs of LLVY chemotrypsin-like activity % in the (**F**). Heart and (**G**). PBMCs expressed as fold change of young adult (13–14 weeks of age) C57BL/6J mice after four doses of Cfz (*n* = 5 in Contol and *n* = 7 in Cfz groups). (**H**). Myocardial MDA content (in mmol/mg protein, *n* = 5 per group). (**I**,**J**). Representative M-Mode images and graph of FS% in the four-dose protocol (*n* = 5 in contol and *n* = 7 in Cfz groups). Data are presented as mean ± SEM. Two-tailed, unpaired *t*-test, * *p* < 0.05, ** *p* < 0.01, *** *p* < 0.005 and **** *p* < 0.001.

Our first step was to investigate the proteasome activity in heart and peripheral blood mononuclear cells (PBMCs) of the aged mice (18 months of age) compared to young adult mice (13–14 weeks of age). We found that both in PBMCs and heart, aging leads to a decrease in proteasome activity (LLVY- chemotrypsin-like activity) compared to young adult mice (Supplementary Figure S1). Furthermore, we sought to confirm that advanced age does not mitigate Cfz’s proteasomal inhibition, therefore, we initially addressed the chemotrypsin-like activity in the PBMCs and the myocardium in the treated aged mice. We found that in the two-dose protocol, Cfz significantly reduced LLVY- chemotrypsin-like activity, corresponding to 26S proteasome activity, both in the heart (Figure 1A) and the PBMCs (Figure 1B). Moreover, we observed that malondialdehyde (MDA), a biomarker of lipid peroxidation widely used in cardiovascular diseases [14], was significantly increased in the hearts of the Cfz-treated mice (Figure 1C). Fractional shortening % (FS%) was found to be significantly decreased in the Cfz group compared to its baseline values (Supplementary Tables S1–S3, Figure 1D,E). In the four-dose protocol, Cfz showed a more pronounced phenotype concerning the proteasomal inhibition both in the myocardium (Figure 1F) and the PBMCs (Figure 1G) accompanied by a significant increase in myocardial MDA content (Figure H). Four doses of carfilzomib led to a more denounced decline in FS %, which was found to be significant both compared to baseline values and to the control group (Figure 1I,J).

### 2.2. Carfilzomib Administration (2 Doses Protocol) Reduced AMPKα Phosphorylation and Increased Bip Expression, without Increasing PP2A Activity

**Figure 2:**
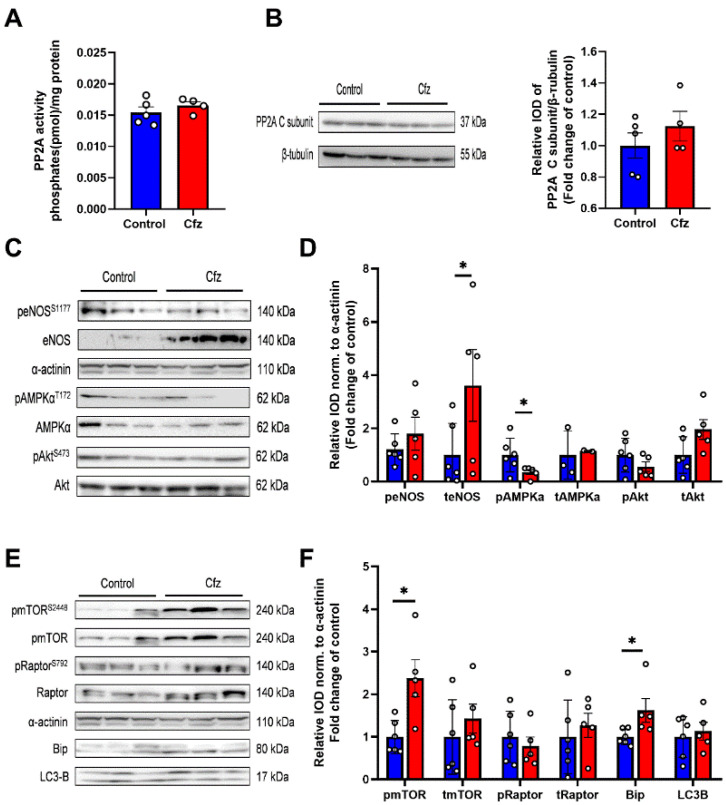
Two doses of carfilzomib decreased AMPKα phosphorylation and upregulated eNOS and Bip expression and mTOR phosphorylation. Graphs of PP2A (**A**). Activity (in pmol/mg protein; *n* = 5 per group) and (**B**). Εxpression (fold change of control). (**C**). Representative western blots and (**D**). Relative densitometry analysis of phosphorylated and total eNOS, AMPKα, and Akt levels normalized to α-actinin. (**E**) Representative western blots and (**F**). Relative densitometry analysis of phosphorylated and total mTOR, Raptor, and total Bip and LC3B normalized to α-actinin (*n* = 6 per group). All protein targets were run on the same gradient SDS page gel. Data are presented as mean ± SEM. Two-tailed, unpaired *t*-test, * *p* < 0.05.

In order to investigate the molecular signaling in the two-dose Cfz protocol, we initially explored PP2A activity and expression in the myocardium, which was previously found to be the key mediator of Cfz’s cardiotoxicity in the young adult mice [6]. We observed that in contrast to the young adult mice, two doses of Cfz did not increase PP2A activity (Figure 2A) and did not upregulate PP2A expression (Figure 2B). Concerning molecular signaling, Cfz led to an upregulation of endothelial nitric oxide synthase (eNOS) expression and decreased AMP-activated protein kinase A (AMPKα) phosphorylation (Figure 2C,D). Moreover, Cfz increased both mammalian target of rapamycin (mTOR) phosphorylation and endoplasmic reticulum (ER) stress marker-binding immunoglobulin protein (Bip) expression, without inhibiting autophagy’s surrogate marker microtubule-associated proteins 1A/1B light chain 3B (LC3B) (Figure 2E,F). Therefore, it seems that two doses of carfilzomib can lead to decreased AMPKα phosphorylation as observed previously in the young adult mice [6], but, additionally, it can lead to increased unfolded protein response as indicated by increased Bip expression in the aged myocardium. Therefore, AMPKα decreased phosphorylation and Bip upregulation can serve as early markers of the Cfz-induced cardiotoxicity in the aged heart.

### 2.3. Carfilzomib Administration (Four-Dose Protocol) Increased PP2A Activity, Reduced AMPKα Phosphorylation and Increased Bip and LC3B-Depedendent Autophagy

**Figure 3:**
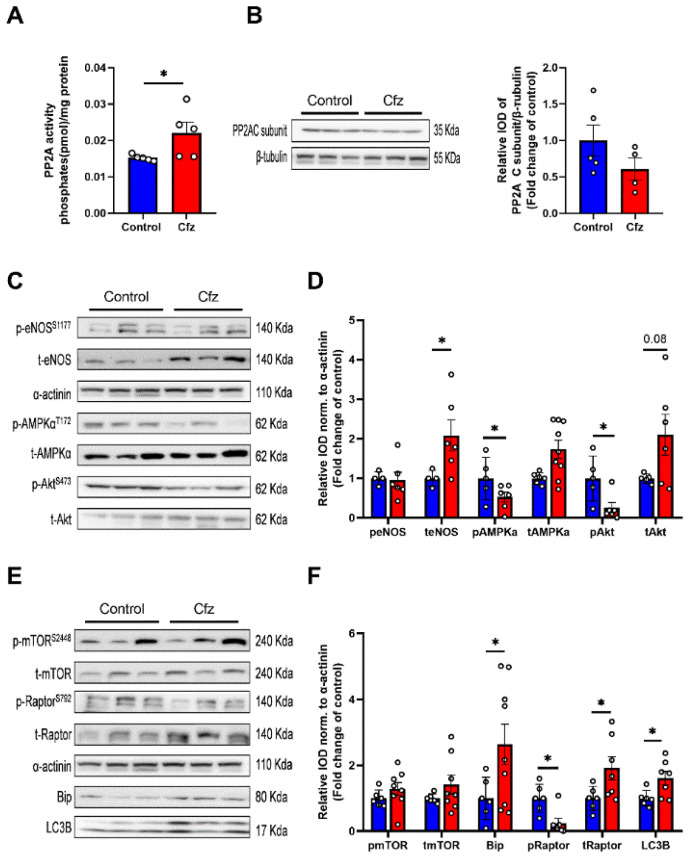
Four doses of carfilzomib increased PP2A activity, decreased AMPKα phosphorylation, and upregulated eNOS, Bip, Raptor, and LC3B expression. Graphs of PP2A (**A**). Activity (in pmol/mg protein; (*n* = 5 per group)) and (**B**). Expression (fold change of control). (**C**). Representative western blots and (**D**). Relative densitometry analysis of phosphorylated and total eNOS, AMPKα, and Akt levels normalized to α-actinin. (**E**). Representative western blots and (**F**) Relative densitometry analysis of phosphorylated and total mTOR, Raptor, and total Bip and LC3B normalized to α-actinin (*n* = 4–6 per group). All protein targets were run on the same gradient SDS page gel. Data are presented as mean ± SEM. two-tailed, unpaired *t*-test, * *p* < 0.05.

Subsequently, we focused on the four-dose protocol, and we investigated the activity of PP2A in the myocardium. We observed that in line with the young adult mice, four doses of Cfz increased PP2A activity (Figure 3A) and did not upregulate PP2A expression (Figure 3B). Concerning the molecular signaling, Cfz upregulated eNOS expression, decreased AMPKα phosphorylation (Figure 3C,D), and showed a trend of increase in Akt expression versus controls (*p* = 0.08). Concerning ER stress and autophagic signaling, Cfz increased both Bip, LC3-B, and Raptor expression while decreased Raptor phosphorylation (Figure 3E,F). Thus, our results demonstrate that Cfz-mediated AMPKα dephosphorylation and increased Bip expression, as observed both in the two- and four-dose protocol is a key mediator of the decline of LV function induced by Cfz. Interestingly, Cfz increased PP2A activity only in the four-dose protocol, indicating that maybe prolonged Cfz administration is required for PP2A activation in the aged mice.

### 2.4. Metformin Did Not Interfere with Carfilzomib’s Proteasome Inhibitory Activity, Restored MDA Levels in the Myocardium, and Maintained LV Function in the Two Doses Protocol

Since we identified that in the aging model, AMPKα dephosphorylation is a key driver of the cardiac phenotype, we sought to investigate whether Met, a clinically used anti-hyperglycemic agent and a known AMPKα activator, can reverse the cardiotoxicity in vivo, as previously reported in the young adult mice [6]. On a translational aspect, we first investigated whether Met hampered Cfz’s proteasome inhibitory capacity in the heart and PBMCs. We found that Met administration did not affect Cfz’s proteasome inhibitory potential neither in the heart nor in the PBMCs of the treated mice in the two-dose protocol (Supplementary Figure S2A,B). Met administration did not alter blood glucose levels (Supplementary Figure S2C). Considering cardiac oxidative stress, we found that only Cfz could statistically significantly increase MDA content in the myocardium, while coadministration with Met maintained MDA levels similar to controls (Supplementary Figure S2D). Concerning the LV function of the mice, only Cfz led to a statistically significant decrease in FS% compared to their baseline values, while no statically significant differences were found among the other groups regarding the baseline and endpoint values (Supplementary Figure S2E,F). Therefore, it seems that Met in the two-dose protocol can successfully mitigate the early mild cardiotoxic phenomena induced by Cfz.

### 2.5. Metformin Mitigated Early Cfz Cardiotoxicity in the Two-Dose Regimen, through Induction of AMPKα Signaling and Synergistic Increase in LC3B

Subsequently, we sought to investigate the molecular mechanism of Met’s cardioprotective potential. Cfz and Met were not found to modulate PP2A expression or activity in the myocardium (Supplementary Figure S3A,B), whereas Met alone and after coadministration with Cfz was found to increase eNOS expression, AMPKα phosphorylation and restored Bip expression, which was increased in the Cfz group. Cfz and Met coadministration favorably decreased Akt and Raptor phosphorylation and LC3B expression (Supplementary Figure S3C–F). It is therefore evident that Met can modulate AMPKα and endoplasmic reticulum (ER) stress marker Bip, synergistically inducing LC3B-dependent autophagy in the myocardium and providing cardioprotection against early Cfz cardiotoxicity.

### 2.6. Metformin Did Not Interfere with Carfilzomib’s Proteasome Inhibitory Potential and Maintained LV Function in the Four-Dose Protocol, Independently of Myocardial Oxidative Stress

**Figure 4:**
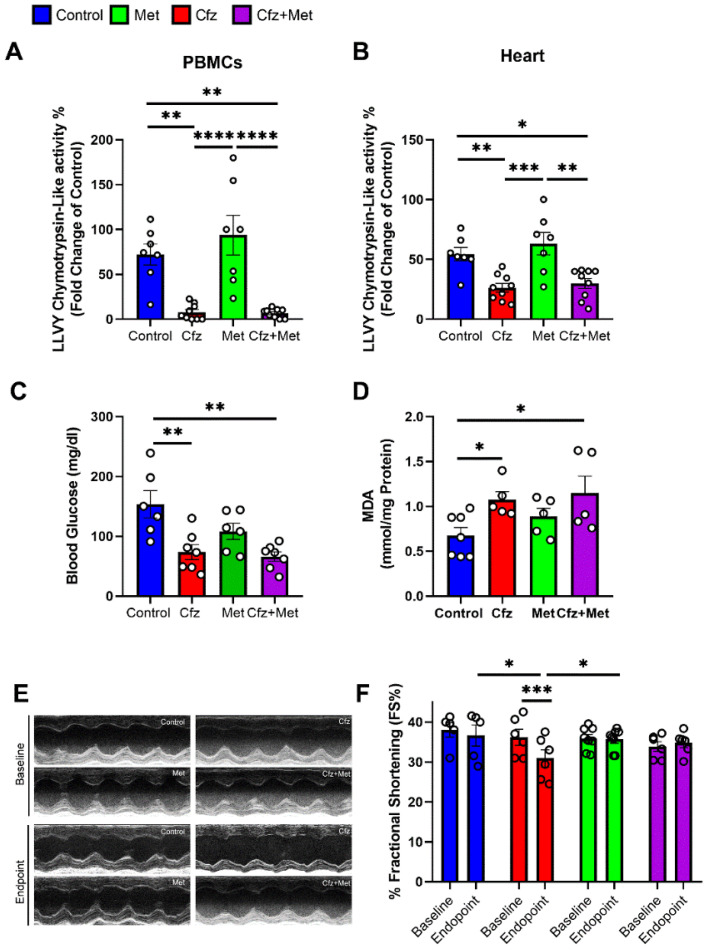
Metformin in the four-dose protocol could successfully protect against Cfz cardiotoxicity without interfering with Cfz proteasome inhibitory activity and independently of myocardial oxidative stress. Graphs of LLVY chemotrypsin-like activity % in the (**A**). PBMCs and (**B**). Heart expressed as fold change of young adult (13–14 weeks of age) C57BL/6J mice after four doses of Cfz (*n* = 7–9 per group). (**C**). Fasting blood glucose levels (mg/dL). (**D**) Myocardial MDA content (in mmol/mg protein, *n* = 5–7 per group). (**E**,**F**). Representative M-Mode images and graph of fractional shortening (FS%) in the four-dose protocol (*n* = 5–8 per group). Data are presented as mean ± SEM. One-Way ANOVA, Tukey post-hoc test, * *p* < 0.05, ** *p* < 0.01, *** *p* < 0.005, **** *p* < 0.001.

After identifying the cardioprotective potential of Met in the early phase of Cfz cardiotoxicity, we focused on the more clinically translational protocol of four doses of Cfz. Initially, we tested whether Met interferes with Cfz proteasome inhibitory potential, and we found that both in heart and PBMCs, Cfz alone and in combination with metformin maintains its proteasome inhibitory activity (Figure 4A,B). Since Met is a clinically used anti-hyperglycemic drug, we investigated the blood glucose levels in the treated mice. Surprisingly we found that Cfz and not Met led to a decrease in fasting blood glucose alone and in the Cfz + Met coadministration group, while Met did not cause a hypoglycemic effect at the selected dose (Figure 4C). Moreover, concerning the myocardial oxidative stress, only Cfz increased MDA content (Figure 4D). Importantly, concerning the LV function, four doses of Cfz led to a statistically significant decrease in FS% compared to the group’s baseline values and compared to control and Met groups, while Met coadministration maintained FS% in the range of the control group, as observed in the Cfz + Met group (Supplementary Tables S4–S6, Figure 4E,F). The latter data showed that coadministration of Cfz and Met could serve as a potent prophylactic regimen, whereas Met does not interfere with Cfz’s antitumor activity in the in vivo model of aging.

### 2.7. Coadministration of Cfz with Metformin Reduced Myocardial PP2A Activity, Partially Restored Bip Expression and Increased AMPKα Phosphorylation Inducing LC3B-Dependent Autophagy in the Aged Myocardium

**Figure 5:**
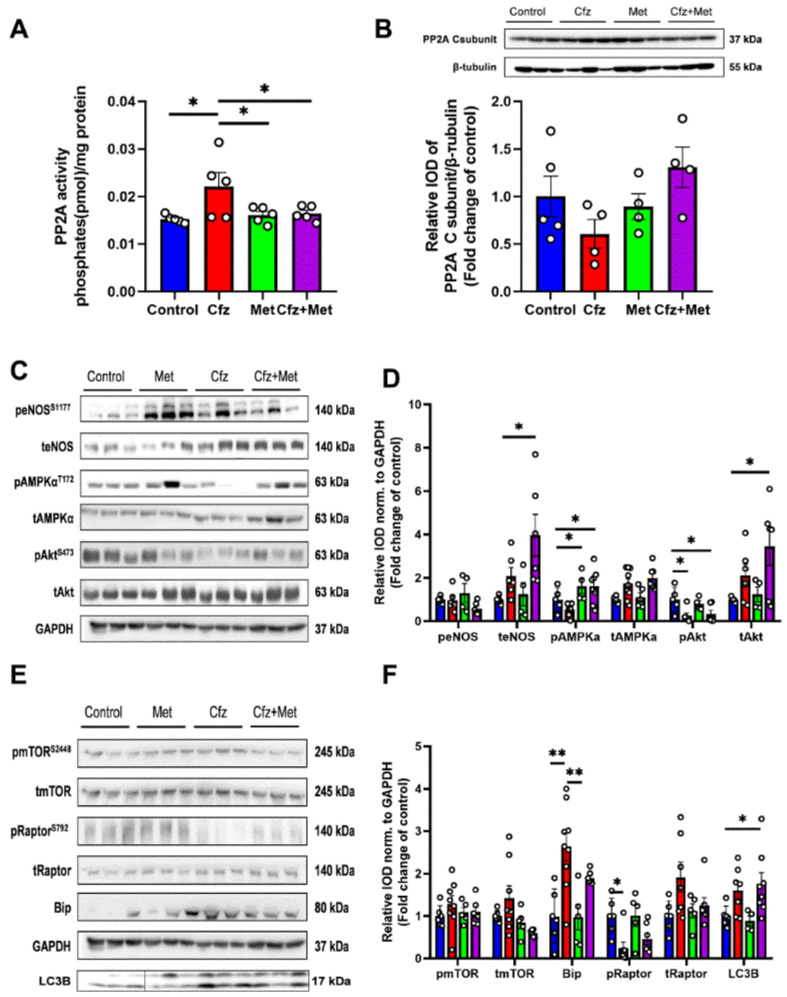
Metformin protected against Cfz cardiotoxicity through increased AMPKα phosphorylation, partial restoration of Bip expression, and synergistic induction with carfilzomib of LC3B-dependent autophagy in the four-dose protocol. Graphs of PP2A (**A**). Activity (in pmol/mg protein; *n* = 5 per group) and (**B**) Expression (fold change of control). (**C**). Representative western blots and (**D**). Relative densitometry analysis of phosphorylated and total eNOS, AMPKα, and Akt levels normalized to Glyceraldehyde 3-phosphate dehydrogenase (GAPDH). (**E**). Representative western blots and (**F**). Relative densitometry analysis of phosphorylated and total mTOR, Raptor, and total Bip and LC3B normalized GAPDH (*n* = 5–7 per group). All protein targets were run on the same gradient SDS page gel. Data are presented as mean ± SEM. One-way ANOVA, Tukey’s post-hoc test, * *p* < 0.05, ** *p* < 0.01.

Subsequently, we initially assessed myocardial PP2A activity and expression. We found that Cfz increased PP2A activity, while Met decreased PP2A activity in the myocardium in the coadministration group. PP2A expression was unchanged between study groups (Figure 5A,B). Met increased AMPKα phosphorylation alone and in combination with Cfz, whereas when co-administered with Cfz partially decreased Bip expression and increased teNOS, tAkt, and LC3B expression. Cfz decreased Akt phosphorylation both alone as well as in the coadministration group, an effect not reversed by Met (Figure 5C–F). Therefore, Met coadministration with Cfz led to the induction of AMPKα/LC3B-dependent autophagy and a partial modulation of ER stress marker Bip in the aged myocardium leading to cardioprotection in the translational model of Cfz cardiotoxicity in aged mice.

## 3. Discussion

In the current work, we investigated the mechanisms of Cfz-induced cardiotoxicity in the aged myocardium, as well as we sought to identify the possible cardioprotective potential of Met. To the best of our knowledge, this is the first time that a model of Cfz-induced cardiotoxicity has been attempted in an in vivo model of aging. We have shown herein that Cfz led to dose-dependent cardiotoxicity in the aged myocardium, as shown by the decreased FS% compared to the young adult controls. Moreover, Cfz led to decreased phosphorylation of AMPKα in the aged myocardium. In contrast to the young adult mice, we observed a Cfz-dependent increase in chaperone Bip expression, a marker of elevated unfolded protein response [15]. We also demonstrated herein that, in compliance with the young adult mice, PP2A phosphatase activity is elevated in the myocardium of the aged mice after four doses of Cfz, which could be associated with Akt and AMPKα dephosphorylation and the manifestation of cardiotoxicity in vivo. Met, presented as a potent prophylactic therapy, as it increased AMPKα phosphorylation and decreased PP2A phosphatase activity in the myocardium, while when administered with Cfz, it induced a synergistic induction of autophagy and maintenance of LV function in vivo.

Aging is an independent risk factor for the manifestation of cardiotoxicity by chemotherapeutics [16]. In terms of MM, this cofounder plays a crucial role, as MM is considered an elderly disease and afflicts individuals >65 years of age [10]. Moreover, advanced age is implicated with molecular and physiological alterations in the myocardium [13,17]. Proteasome activity is found to be decreased in the aged myocardium [18], alongside PP2A phosphatase activity in the heart [19]. Moreover, the deterioration of the Proteasome-Ubiquitin system (UPS) is a key driver of dysregulation of autophagy in the aged myocardium [20], which was also observed in our experiments (Supplementary Figure S1). All these entangled molecular in the aged myocardium contribute to the manifestation of age-related cardiomyopathies.

It is already recognized that the most life-threatening adverse effects of the irreversible proteasome inhibitor Cfz are cardiovascular-system-related, and HF stands among the most serious complications of Cfz, often leading to the discontinuation of the therapy [2,3,4,21]. Identifying this imminent threat in managing MM, we previously established a translational in vivo model of carfilzomib cardiotoxicity in young adult mice. The observed phenotype was orchestrated by PP2A-dependent dephosphorylation of AMPKα, leading to impairment of myocardial autophagy. However, the investigation of Cfz cardiotoxicity in a young adult murine model lacked direct translational value as most of these adverse events are noted in elderly individuals, in which myocardial homeostasis mechanisms are malfunctioning. Herein, in contrast to the young adult murine model, we found that during early Cfz cardiotoxicity (two-dose protocol), PP2A does not play such an important role. This can be justified by the fact that it is previously shown that PP2A phosphatase activity is reduced in an age-dependent manner [19]. Therefore, the induction of PP2A activity upregulation by carfilzomib seems to be dose-dependent in the aged myocardium. Despite the absence of increased PP2A activity in the two-dose protocol, we did observe a decreased phosphorylation of AMPKα and an increased expression of Bip, with the latter not being previously observed in the young adult mice. Bip is a protein chaperone, which is an endogenous sensor of unfolded proteins, acting as a carrier of the proteins to the endoplasmic reticulum for repair [22,23]. Aging is a known mediator of increased unfolded protein generation, leading to an upregulation of Bip and, therefore, to increased unfolded protein response [24]. Excessive and unresolved unfolded protein response can subsequently lead to ER stress, which is an end-effector of the manifestation of age-dependent cardiomyopathies. Moreover, unfolded protein response and ER stress are known to mediate the induction of autophagy as an endogenous compensatory mechanism in managing faulted proteins and maintaining cellular homeostasis [25]. The latter can justify that despite the fact that AMPKα phosphorylation is decreased in the Cfz-treated aged myocardium, LC3B, a surrogate marker of LC3B-depdendent autophagy is found to be increased, indicating a Bip-dependent and AMPKα-independent induction of autophagy in the Cfz-treated aged myocardium in our protocol. This seems to be in contrast to what we previously found in young adult mice, in which Cfz led to a decrease in LC3B-dependent autophagy in the myocardium [6], while resembles the molecular signaling observed in the Cfz-treated aortas [26], in which ER stress markers calnexin and Bip were found to be elevated leading to increase in LC3B-dependent autophagy. This can be attributed to the increased sensitivity of the vascular musculature to unfolded protein response compared to the myocardium in the young adult mice [27]. Considering the decreased AMPKα phosphorylation, unfolded protein response and ER stress are known to inhibit AMPKα [28,29]. Therefore, increased Bip expression can be the key driver of AMPKα reduced phosphorylation, while PP2A phosphatase might play a lesser role in the aged myocardium.

Concerning Akt/eNOS pathway, referred also as an endogenous cardioprotective axis [30], we found that Cfz increased eNOS expression in the aged myocardium, an effect not observed in the young adult mice. It is previously shown that Cfz modulated eNOS expression and administration of boronate-based agent MLN-273, which is also an irreversible proteasome inhibitor, in pigs resulted in increased eNOS expression that was uncoupled from NO production and was associated with increased coronary artery oxidative stress, functional and structural cardiac deficits leading to LV dysfunction [31,32,33]. Herein and in compliance with literature, we observed that despite the fact that Cfz led to an increased eNOS expression, activatory phosphorylation of the enzyme at S1177 was unchanged among groups, indicating that eNOS upregulation might be related to oxidative stress-inducing phenomena due to uncoupling rather than eNOS activation, which in turn might justify the increased myocardial MDA content in Cfz-treated groups. Regarding the even greater upregulation of eNOS in the Cfz + Met groups, both in the two- and four-dose protocols can be justified by the already known effect of Met on upregulating eNOS expression on a transcriptional level [34], which could act synergistically with Cfz.

Additionally, we found that in Cfz + Met groups, Akt phosphorylation was decreased in contrast to what we previously reported in the young adult mice [6], and this is in agreement with the knowledge that Akt phosphorylation and regulation play a different role in cardiac homeostasis in the aged myocardium and are associated with cardiac hypertrophy phenomena [35]. Increased transcriptional regulation of Akt via oxidative stress and increased Akt phosphorylation are associated with activation of hypertrophy-related genes (i.e., Myc, FOXO, Bax, FasL), exacerbation of hypertrophy and HF [35]. Therefore, reduced Akt phosphorylation in the Cfz + Met groups might be a rather cardioprotective effect of the drug combination and can be associated with the improvement of LV function.

Despite the fact that the upstream effectors of AMPKα phosphorylation seem to be different in the young and the aged myocardium, AMPKα seems to play an important role in the manifestation of Cfz cardiotoxicity in aging. AMPKα is a pivotal kinase implicated in numerous cellular processes, such as cellular viability, differentiation, bioenergetics, and autophagy [36,37]. Concerning autophagy, AMPKα is known to inhibit mTORC2 complex, an inhibitor of autophagy, and phosphorylate Raptor leading to increased autophagic flux [38]. Restoration of AMPKα and autophagy in the aged myocardium seems to be of utmost significance, as compounds such as Met and resveratrol, well-recognized AMPKα activators [7,39,40], are proven to reverse cellular senescence and age-related cellular deficits [41]. Taking under consideration that Met is a clinically applicable AMPKα activator, which acts through intracellular AMP accumulation via complex I inhibition [7], and since we have previously shown that Met exerted a prophylactic potential in the young adult mice [6], we tried to confirm our previous findings in the aged mice, proving in parallel that AMPKα is crucial for the cardioprotection against Cfz cardiotoxicity also in the aged myocardium. Indeed, Met hampered Cfz-induced decrease in LV function, as shown by FS% both in the two- and four-dose protocols.

**Figure 6:**
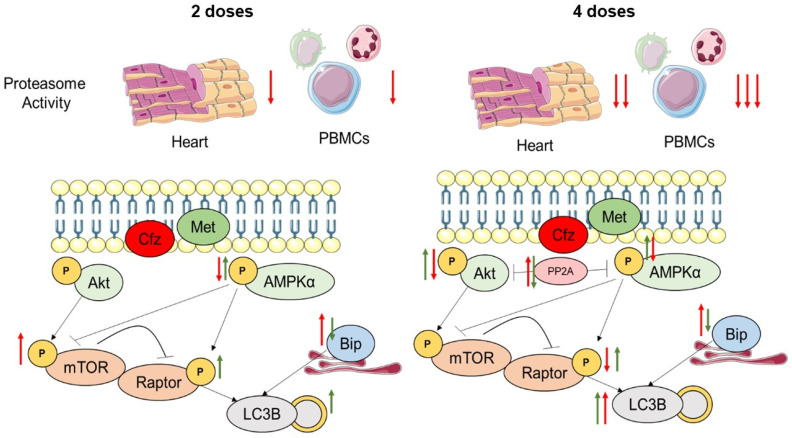
Proposed mechanism of carfilzomib-induced cardiotoxicity and metformin prophylactic effect in the aged myocardium. Red arrows correspond to carfilzomib’s effect, and green arrows correspond to metformin’s effect. Arrows indicate activation/induction, and blunted lines indicate inhibition. Akt, protein kinase B; AMPKα, AMP-activated kinase α; Bip, binding immunoglobulin protein; Cfz, carfilzomib; Met, metformin; mTOR, mammalian target of rapamycin; LC3B, microtubule-associated proteins 1A/1B light chain 3B; PP2A, protein phosphatase 2 A; Raptor, regulatory-associated protein of mTOR.

Met was also found to restore Bip expression to control levels in the two-dose protocol, while partially restored Bip expression in the four-dose protocol, possibly due to the sustained proteasome inhibition by Cfz and therefore excessive accumulation of unfolded proteins. Our data are in agreement with the literature, as it is previously shown that Met can modulate ER stress and unfolded protein response in an AMPKα-dependent downregulation of ER stress markers such as Parkin and miR-132, in the myocardium and in other organs [42,43,44]. However, to the best of our knowledge, it is the first time that the down-regulatory effect of Met specifically on Bip is presented, indicating that Met could mitigate unfolded protein response. Finally, in the coadministration group, Cfz and Met synergistically induced LC3-B-dependent autophagy via a complementary activation of ER stress and AMPKα. As previously mentioned, induction of autophagy in the aged myocardium is already recognized as a cardioprotective approach, and thus, the synergistic induction of autophagy in the Cfz + Met group could reason with the improved LV function and can substantiate the establishment of Met as prophylactic therapy in aged individuals (Figure 6). A limitation of the study is that the mechanistic insight herein refers to the whole myocardial tissue and not to a specific cellular population. It is not clear if carfilzomib’s cardiotoxic phenomena are mediated directly on the cardiomyocytes or whether other myocardial cell populations (i.e., endothelial cells, fibroblasts, or pericytes) are involved in the pathogenesis of the cardiotoxicity. However, more complex in vitro primary cell experiments are required to resolve this issue.

Noteworthily, in the Cfz-treated mice in the four-dose protocol, we observed that Cfz induced a statistically significant decrease in fasting blood glucose, an effect not observed after Met administration. This effect seems to be age related, as it was not observed in young adult mice [45]. In clinical trials concerning Cfz, MM patients are shown to present hyperglycemic events, which are attributed to the coadministration of the drug with dexamethasone within the KRd regimen [46]. Hypoglycemia in Cfz-treated patients is reported in an open-label phase 2 trial of carfilzomib-cyclophosphamide-dexamethasone in elderly patients with newly diagnosed MM; however, it was manifested at low prevalence [47]. Despite the fact that the hypoglycemic effect of Cfz could be species-and age-specific, it could be related to the modified urinary glucose excretion due to Cfz-induced nephrotoxicity [45]. Nevertheless, this new finding concerning the Cfz effect of blood glucose requires further investigation.

## 4. Materials and Methods

### 4.1. Animals

Sixty male C57Bl/6J mice were used for the conduction of this study. Mice were naturally allowed to age up to 17–18 months of age, which is considered to be an appropriate age for the simulation of elderly individuals. The age of 18 months corresponds to a human equivalent age of 56–69 years, which is in line with the median age of the patients with MM, which is approximately 66 years of age [48]. Twelve additional male C57Bl/6J mice 13–14 weeks of age were used as young adult controls for the proteasome activity assessment in vivo. Mice were bred and housed in the Animal Facility of Biomedical Research Foundation Academy of Athens. All animal experiments were carried out in accordance with the “Guide for the care and use of Laboratory animals”, and experiments were approved by the ethics committee (approval no: 182464;14-05-2019). Animals were housed and maintained in SPF cages (10 per cage; 25 ± 1 °C) at least for one week before the experiments, according to ARRIVE guidelines [49,50]. Subsequently, mice were randomized as follows: Two-dose protocol: i. Control (NaCl 0.9%), ii. Cfz (8 mg/kg), iii. Met (140 mg/kg), iv. Cfz + Met (8 mg/kg, 140 mg/kg, respectively) for 2 days [6], *n* = 5–7 per group. Four-dose protocol: i. Control (NaCl 0.9%), ii. Cfz (8 mg/kg), iii. Met (140 mg/kg), iv. Cfz + Met (8 mg/kg, 140 mg/kg, respectively) for 6 days [6], *n* = 5–7 per group. NaCl and Cfz were injected intraperitoneally daily on the 2-dose protocol and on alternate days in the 4-dose protocol, while Met was administered by oral gavage daily, for 2 and 6 days, during the 2- and 4-dose protocols, respectively. Drug regimens were based on our previous study addressing the cardiotoxicity of carfilzomib; therefore, it is already established that by both a functional and a mechanistic aspect, the 4-dose carfilzomib administration can successfully mimic the clinical events observed in treated MM individuals [6]. At the end of the experiments, animals were anesthetized by ketamine (100 mg/kg) and were euthanized by cervical dislocation. Blood samples and myocardial tissue were collected. Blood samples were used for the determination of the chymotrypsin-like activity of the 26S proteasome, while myocardial tissues were used for the assessment of proteasome activity and for molecular and histological analysis.

### 4.2. PBMCs Isolation

PBMCs were isolated using lymphosep (density 1077 g/mL, Biosera, Nuaille, France) from freshly collected blood; blood was then diluted (1:1 dilution) with phosphate-buffered saline (PBS). The diluted whole blood was carefully layered over the separation medium (1/2 × the volume of the sample), and the two phases were kept separated before the centrifugation. Samples were then centrifuged at 400× *g* for 30 min at 20 °C. PBMCs were collected after aspiration of the plasma and platelets layer; cells were then washed with PBS and processed for proteasome peptidase activity [6].

### 4.3. Measurement of Proteasome Peptidase Activity

For measuring proteasome peptidases activity, dissected heart tissue or isolated PBMCs were lysed on ice in a 26S proteasomes isolation buffer as previously described [6]. Protein content was adjusted with Bradford, and supernatants were immediately used (after the addition of the fluorogenic substrate) to determine the CT-L proteasomal peptidase activity; all measurements were performed in duplicates. Emitted fluorescence was recorded at Spark^®^ Tecan microplate reader (Tecan Group Ltd., Maennedorf, Switzerland) a VersaFluor Fluorometer System (Bio-Rad Laboratories, Hercules, CA, USA) at excitation and emission wavelengths of 380 nm and 460, 350, and 440 nm, respectively. Results are expressed as % ± SEM fold change of 13–14-week-old C57Bl/6J mice used as control samples, referred to as young adult mice in the manuscript.

### 4.4. Echocardiography

Echocardiographic analysis was performed in anesthetized mice with isoflurane (5% in 1 L/min oxygen for induction and 1% for maintenance of anesthesia). Body temperature was kept at 37 °C using a heating system within the handling platform. Transthoracic echocardiography was performed by an experienced sonographer in a blinded manner using the VEVO2100 high-resolution imaging system (VisualSonics®, FujiFilm, Toronto, Canada) equipped with an 18–38 MHz linear-array transducer (MS400). Post-acquisition analysis was performed with the VevoLab Software Version 3.2.0. (VisualSonics®, FujiFilm). Heart rate (HR), left ventricular end-diastole (LVEDD) and left ventricular end-systole diameter (LVESD), left ventricular posterior wall thickness at diastole (LVPWd), fractional shortening (FS; FS% = (LVEDD − LVESD)/LVEDD × 100%), cardiac output (CO, mL/min) and left ventricular radius to left ventricular posterior wall thickness ratio were calculated [51].

### 4.5. Western Blot Analysis

Myocardial tissue powder was extracted with lysis buffer (1% Triton X–100, 20 mM Tris pH 7.4–7.6, 150 mM NaCl, 50 mM NaF, 1 mM EDTA,1 mM EGTA, 1 mM Glycerolphosphatase, 1% SDS, 100 mM phenylmethylsulfonyl fluoride, and 0.1% protease phosphatase inhibitor cocktail). After centrifugation (11,000× *g*, 15 min, 4 °C), supernatants were used for protein analysis, as previously described [30]. The following primary antibodies were used: pmTOR (mammalian target of rapamycin; Ser2448) (D9C2) (Rabbit mAb, #5536), mTOR (Rabbit mAb, #2983), p-Raptor (regulatory-associated protein of mTOR; Ser792) (Rabbit mAb, #2083), Raptor (Rabbit mAb, #2280), p-eNOS (endothelial nitric oxide synthase; Ser1177) (Rabbit mAb, #9570), eNOS (Rabbit mAb, #9572), p-AMPKα (AMP-activated kinase α Thr172) (Rabbit mAb, #2535), AMPKα (Rabbit mAb #5831), p-Akt (protein kinase B, Ser473) (Rabbit mAb, #9271), Akt Antibody (Rabbit mAb, #9272), β-tubulin (Rabbit mAb, #2146), α-actinin (Rabbit mAb #6487), Bip (binding immunoglobulin protein; Rabbit mAb, #3177), LC3B (microtubule-associated protein 1A/1B-light chain 3B, Rabbit mAb, #3868), GAPDH (Glyceraldehyde 3-phosphate dehydrogenase, Rabbit mAb, #5174) (Cell Signaling Technology). PVDF membranes were then incubated with secondary antibodies for 2 h at room temperature [goat anti-mouse (#7076) and goat anti-rabbit HRP (#7074); Cell Signaling Technology, Beverly, MA, USA] and developed using the GE Healthcare ECL Western Blotting Detection Reagents (Thermo Scientific Technologies; Waltham, USA). Relative densitometry was determined using a computerized software package (NIH, Bethesda, USA), and relative ratios were used for statistical analysis [30].

### 4.6. Myocardial MDA Content

Myocardial tissues were pulverized and extracted in Tris-HCl buffer (pH = 7.4), and 70 μL of the supernatant was mixed with 216 μL of N-methyl-2-phenyl-indole (10.3 mM in acetonitrile) and 52.5 μL of 12N HCl. Samples were incubated at 45 °C for 1 h and then centrifuged at 5000× *g* for 5 min at 4 °C. The supernatant was used for the measurement of MDA content (at 540 nm), and myocardial MDA was normalized to protein content [30].

### 4.7. PP2A Activity Assay

PP2A activity was assessed as previously described [6] and according to the manufacturer’s instructions. Briefly, myocardial powders were extracted in an appropriate lysis buffer (20 mM imidazole-HCl. A total of 2 mM EGTA, 2 mM EDTA) supplemented with protease inhibitors. A total of 50 μg of protein was incubated with 40 μL protein A agarose beads and 4 μL of anti-PP2A, C subunit antibody for 2 h at 4 °C. Subsequently, beads were washed 3 times, 60 μL of phosphopeptide were added, and the samples were incubated for 10 min at 30 °C. Finally, the samples were briefly centrifuged, and 25 μL of supernatant were transferred into a microplate, and 100 μL of malachite green were added. Absorbance was measured at 650 nm, and PP2A activity was assessed as μmol/min and normalized to the total protein content of the samples.

### 4.8. Statistical Analysis

Statistical analysis was performed with GraphPad Prism 8 software (Graphpad, San Diego, USA), and results are plotted as mean ± SEM values. Data were analyzed using One-Way ANOVA (Tukey post-hoc test for multiple comparisons) as required, when comparisons refer to more than two groups (i.e., in Figure 4 and Figure 5, Supplementary Figures S2 and S3 and Supplementary Tables S1, S2, S4 and S5). Two-tailed unpaired *t*-test was performed in comparisons referring to two groups Figure 1, Figure 2 and Figure 3and Supplementary Figure S1. *p* values of *p* < 0.05 were considered statistically significant (* *p* < 0.05, ** *p* < 0.01, *** *p* < 0.001). No outliers due to biological diversity were excluded. Samples that did not meet our technical criteria (i.e., low protein content) were not included in the analyses a priori. The confirmation of the absence of outlying values was confirmed by GraphPad Prism analysis software, using the ROUT method and Q = 1%.

## 5. Conclusions

Carfilzomib induces cardiotoxicity in aged mice in an AMPKα-dependent manner, primarily through upregulation of unfolded protein response and secondarily through increased PP2A activity. Coadministration with Met maintained LV function through activation of AMPKα and synergistic induction of autophagy in the aged myocardium. These findings are of great importance for clinical practice. Management of Cfz’s cardiovascular adverse phenomena and especially heart failure is of utmost importance as it is one of the main factors for treatment discontinuation and disease relapse. Despite the fact that the grounds for the prophylactic potential of Met against Cfz’s cardiotoxicity are already set, the confirmation that Met is a safe and well-tolerated prophylactic therapy in the preclinical setting in an aging model can fuel the scientific interest, in terms of clinical research, for the establishment of the prophylactic regimen of Cfz and Met coadministration.

## Data Availability

The data underlying this article are available in the article and in its online Supplementary Material.

## Supplementary Materials

Available online at https://www.mdpi.com/article/10.3390/ijms222010956/s1.
