# Supplementary material for: Elucidating Carfilzomib’s Induced Cardiotoxicity in an In Vivo Model of Aging: Prophylactic Potential of Metformin
Source: Int J Mol Sci. 2021 Oct 11;22(20):10956. doi: 10.3390/ijms222010956 (PMC8537073; doi:10.3390/ijms222010956)
Supplement: Supplementary file 1 [file ijms-22-10956-s001.zip › ijms-1387671-supplementary.pdf]

Elucidating Carfilzomib's induced cardiotoxicity in an in vivo model of aging: prophylactic potential of metformin.

Panagiotis Efentakis[1], Garyfalia Psarrakou[1†], Aimilia Varela[2], Eleni Dimitra Papanagnou[3], Michail Chatzistefanou[1], Panagiota-Efstathia Nikolaou[1], Costantinos H. Davos[2], Maria Gavriatopoulou[4], Ioannis P. Trougakos[3], Meletios Athanasios Dimopoulos[4], Ioanna Andreadou[1*#], Evangelos Terpos[4*].

1. *Laboratory of Pharmacology, Faculty of Pharmacy, National and Kapodistrian University of Athens, Athens, Greece;*
2. *Cardiovascular Research Laboratory, Clinical, Experimental Surgery & Translational Research Center, Biomedical Research Foundation Academy of Athens, Athens, Greece;*
3. *Department of Cell Biology & Biophysics Faculty of Biology, National and Kapodistrian University of Athens, Athens, Greece*
4. *Department of Clinical Therapeutics, School of Medicine, National and Kapodistrian University of Athens, Athens, Greece.*

* *Equally Contributes Senior Authors*
† *In memory of our late colleague Garyfalia Psarrakou*

\# **Correspondence:** Ioanna Andreadou, Ph.D, Faculty of Pharmacy, Panepistimiopolis, Zografou, Athens 15771, Greece; tel: +30 210 7274827; fax: +30 210 7274747; e-mail: jandread@pharm.uoa.gr

Supplemental Tables

Table S1: Echocardiographic analysis of aged mice in the two-dose protocol at baseline

**Table S1**	**Control**	**Cfz**	**Met**	**Cfz+Met**
**P2_Day 0**	**n=7**	**n=6**	**n=5**	**n=6**
**HR**	524.28±7.19	524.33±17.16	488.00±16.24	525.33±10.66
**LVEDD(mm)**	4.06±0.12	4.10±0.05	3.99±0.11	4.03±0.21
**LVESD(mm)**	2.63±0.12	2.59±0.09	2.57±0.11	2.55±0.14
**PWTd (mm)**	0.77±0.01	0.78±0.01	0.76±0.01	0.78±0.01
**FS%**	35.23±1.38	36.86±1.68	35.62±1.77	36.86±1.45
**CO (mL/min)**	24.14±1.16	26.10±1.38	22.46±2.07	25.83±3.58
**r/h**	2.65±0.11	2.63±0.05	2.61±0.11	2.56±0.12

Baseline values of aged mice in the two-dose protocol. Values are presented as mean ± SEM. HR: Heart Rate; LVEDD: Left Ventricular End Diastolic Diameter (mm); LVESD: Left Ventricular End Systolic Diameter (mm); PWTd: Posterior Wall Thickness diastole (mm); FS%: Fractional Shortening %; r/h: ratio of LV radius to PWT-posterior wall thickness.

Table S2: Echocardiographic analysis of aged mice in the two-dose protocol at endpoint

**Table S2**	**Control**	**Cfz**	**Met**	**Cfz+Met**
**Day 2**	**n=7**	**n=6**	**n=5**	**n=6**
**HR**	554.42±16.75	533.33±27.20	507.60±16.88	507.16±11.35
**LVEDD (mm)**	3.72±0.13	4.06±0.11	4.21±0.08	3.86±0.17
**LVESD (mm)**	2.43±0.11	2.84±0.17	2.82±0.07	2.54±0.19
**PWTd (mm)**	0.75±0.01	0.71±0.01	0.74±0.01	0.77±0.01
**FS%**	34.84±1.11	30.40±2.42	33.04±0.45	34.60±2.62
**CO (mL/min)**	21.00±0.92	22.21±1.59	24.99±1.23	20.76±1.73
**r/h**	2.48±0.09	2.84±0.10*†	2.85±0.08*†	2.47±0.11

Endpoint values of aged mice in the two-dose protocol. Values are presented as mean ± SEM. HR: Heart Rate; LVEDD: Left Ventricular End Diastolic Diameter (mm); LVESD: Left Ventricular End Systolic Diameter (mm); PWTd: Posterior Wall Thickness diastole (mm); FS%: Fractional Shortening %; r/h: ratio of LV radius to PWT-posterior wall thickness. One-Way ANOVA, Bonferroni post-hoc analysis, *P<0.05 vs control, †P<0.05 vs Cfz+Met

Table S3: Student's T-Test analysis of Cfz Baseline and Endpoint values in the two-dose protocol that were statistically significantly affected.

Table S3	
**Baseline vs Endpoint**	**p value**
PWTd (mm)	0.0047
FS%	0.0274

Student's T-Test analysis of Cfz Baseline and Endpoint values that were statistically significantly affected. PWTd: Posterior Wall Thickness diastole (mm); FS%: Fractional Shortening %. Student's T-Test, paired, two-tailed.

Table S4: Echocardiographic analysis of aged mice in the four-dose protocol at baseline

Table S4	Control	Cfz	Met	Cfz+Met
**P6_ Day 0**	**n=5**	**n=6**	**n=8**	**n=6**
**HR**	541.00±4.47	528.16±19.88	563.75±10.16	542.33±15.88
**LVEDD (mm)**	3.90±0.06	4.00±0.18	3.77±0.13	3.84±0.11
**LVESD (mm)**	2.41±0.07	2.55±0.14	2.42±0.11	2.53±0.06
**PWTd (mm)**	0.76±0.01	0.75±0.01	0.75±0.01	0.76±0.03
**FS%**	38.00±1.82	36.20±1.97	35.86±1.00	33.87±1.21
**CO (mL/min)**	24.57±1.61	24.08±2.72	22.65±1.43	21.94±1.44
**r/h**	2.56±0.08	2.67±0.13	2.50±0.08	2.51±0.10

Baseline values of aged mice in the four-dose protocol. Values are presented as mean ± SEM. HR: Heart Rate; LVEDD: Left Ventricular End Diastolic Diameter (mm); LVESD: Left Ventricular End Systolic Diameter (mm); PWTd: Posterior Wall Thickness diastole (mm); FS%: Fractional Shortening %; r/h: ratio of LV radius to PWT-posterior wall thickness.

Table S5: Echocardiographic analysis of aged mice in the four-dose protocol at endpoint

Table S5	Control	Cfz	Met	Cfz+Met
P6_Day 6	n=5	n=6	n=8	n=5
HR	527.40±38.15	506.00±20.01	561.00±24.18	536.77±11.82
LVEDD (mm)	3.78±0.05	3.74±0.14	3.68±0.10	3.71±0.18
LVESD (mm)	2.40±0.11	2.59±0.15	2.37±0.09	2.42±0.13
PWTd (mm)	0.75±0.01	0.74±0.01†	0.74±0.01†	0.77±0.01
FS%	36.63±2.64	30.94±2.14*♯	35.67±0.93	34.77±1.13
CO (mL/min)	21.64±1.99	17.94±1.87	21.09±1.05	20.47±2.12
r/h	2.49±0.03	2.54±0.11	2.50±0.06	2.40±0.12

Endpoint values of aged mice in the four-dose protocol. Values are presented as mean ± SEM. HR: Heart Rate; LVEDD: Left Ventricular End Diastolic Diameter (mm); LVESD: Left Ventricular End Systolic Diameter (mm); PWTd: Posterior Wall Thickness diastole (mm); FS%: Fractional Shortening %; r/h: ratio of LV radius to PWT-posterior wall thickness. One-Way ANOVA, Bonferroni post-hoc analysis, *P<0.05 vs Control, †P<0.05 vs Cfz+Met, ♯P<0.05 vs Met

Table S6: Student's T-Test analysis of Cfz Baseline and Endpoint values in the four-dose protocol that were statistically significantly affected.

Table S6	
Baseline vs Endpoint	p value
FS%	0.0010

Student's T-Test analysis of Cfz Baseline and Endpoint values that were statistically significantly affected. FS%: Fractional Shortening %. Student's T-Test, paired, two-tailed.

Supplemental Figures

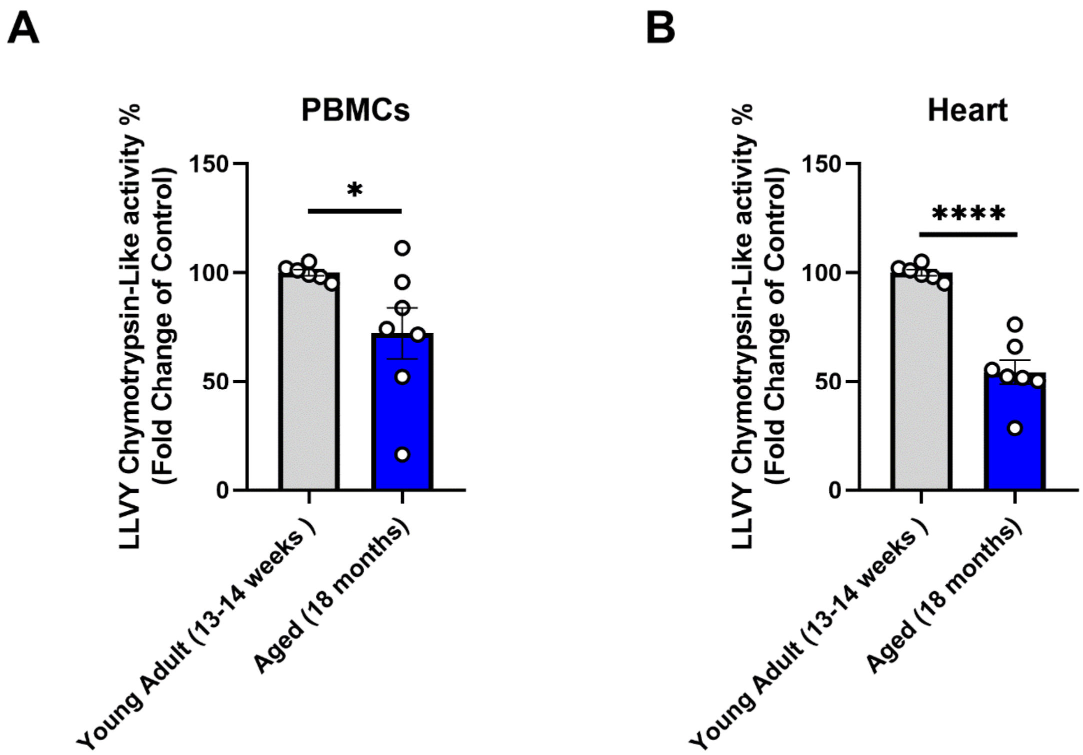

Figure S1. Aged mice present reduced LVVY proteasome activity compared to young adult mice both in heart and PBMCs. Graphs of LLVY Chemothrypsin-Like activity % in the A. PBMCs and B. Heart expressed as Fold change of young adult (13 weeks of age) C57BL/6J mice. All data are presented as mean ± SEM. Two Tailed unpaired T-Test; *P<0.05, ****P<0.001. PBMCs, peripheral blood mononuclear cells

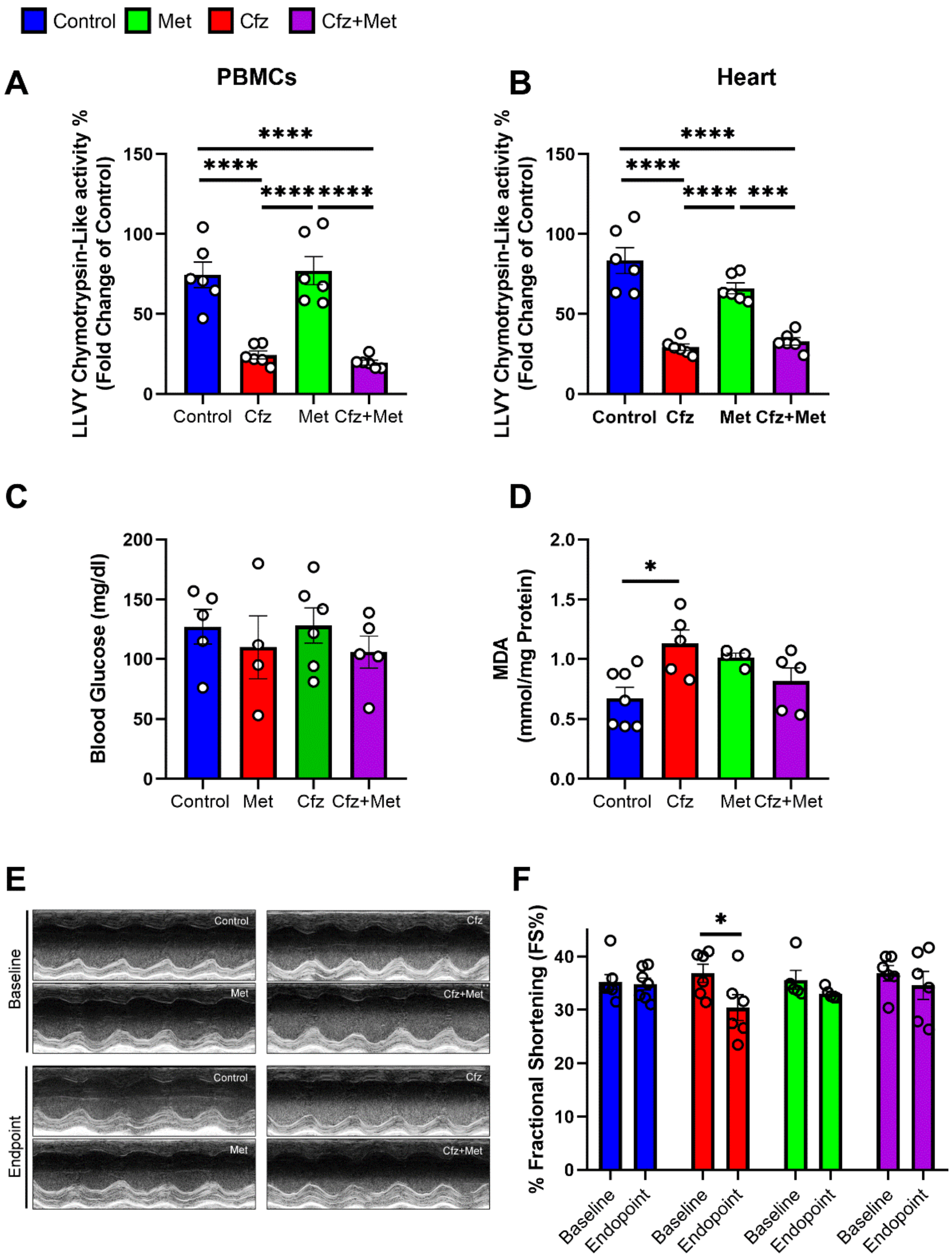

Figure S2: Metformin in the two doses protocol can successfully mitigate the early mild cardiotoxic phenomena induced by Cfz. Graphs of LLVY Chemothrypsin-Like activity % in the **A.** PBMCs and **B.** Heart expressed as Fold change of young adult (13 weeks of age) C57BL/6J mice after two doses of Cfz (n=6 per group). **C.** Fasting Blood glucose levels (mg/dl). **D.** Myocardial MDA content (in mmol/mg protein, n=4-7 per group). **E, F**. Representative M-Mode images and graph of Fractional Shortening (FS%) in the two doses protocol (n=5-7 per group). All data are presented as mean ± SEM. One-way ANOVA, Tukey's post-hoc test, *P<0.05, ***P<0.005, ****P<0.001.

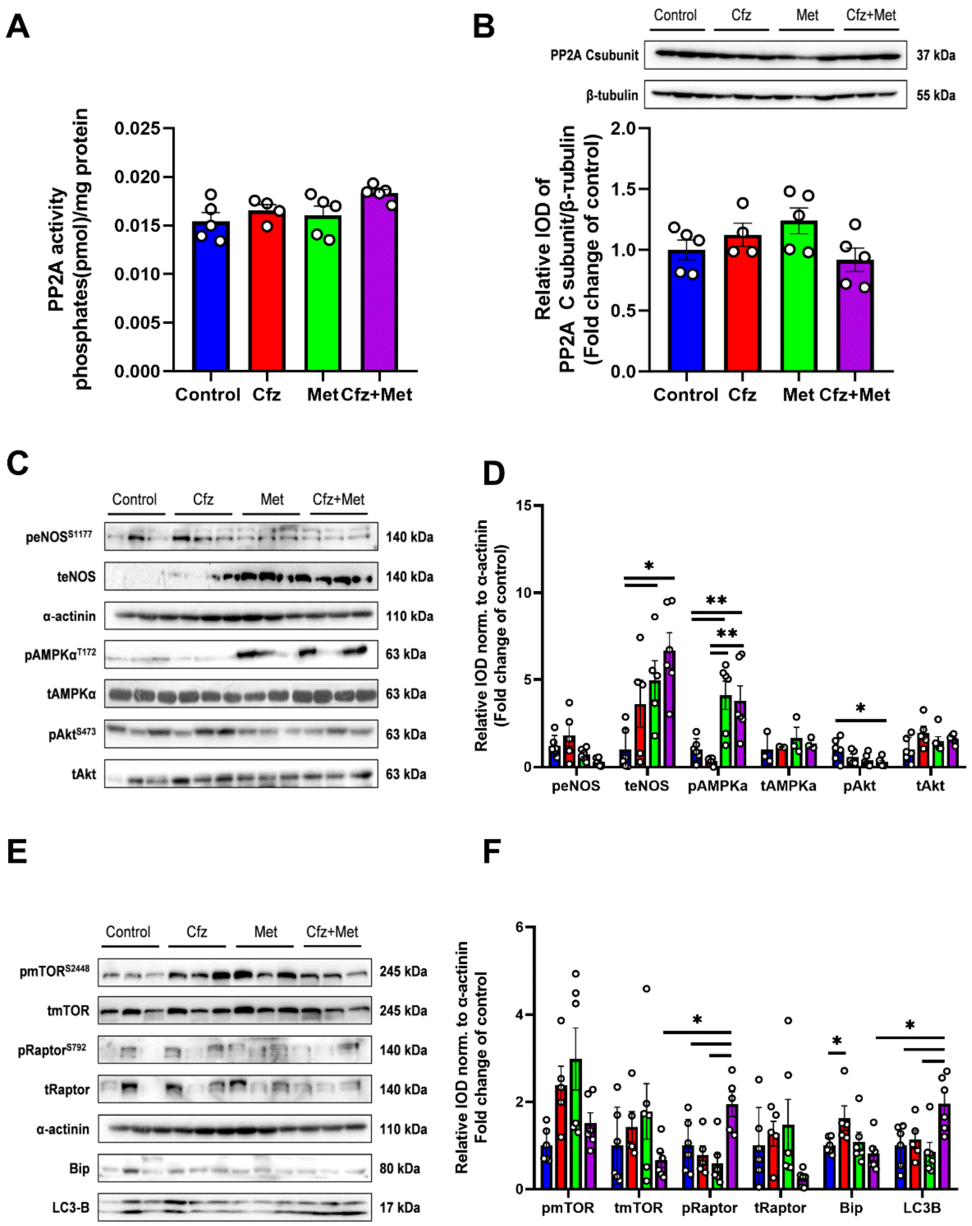

Figure S3: Metformin protects against early Cfz cardiotoxicity through AMPKα phosphorylation, restoration of Bip expression and synergistic induction of LC3B-dependent autophagy. Graphs of PP2A **A.** activity (in pmol/mg protein) and **B.** expression (fold change of control) (n=5 per group). **C.** Representative western blots and **D.** Relative densitometry analysis of phosphorylated and total eNOS, AMPKα and Akt levels normalized to α-actinin. **E.** Representative Western blots and **F.** Relative densitometry analysis of phosphorylated and total mTOR, Raptor and total Bip and LC3B normalized to α-actinin (n=5-6 per group). All data are presented as mean ± SEM. One-way ANOVA, Tukey's post-hoc test, *P<0.05, **P<0.01.

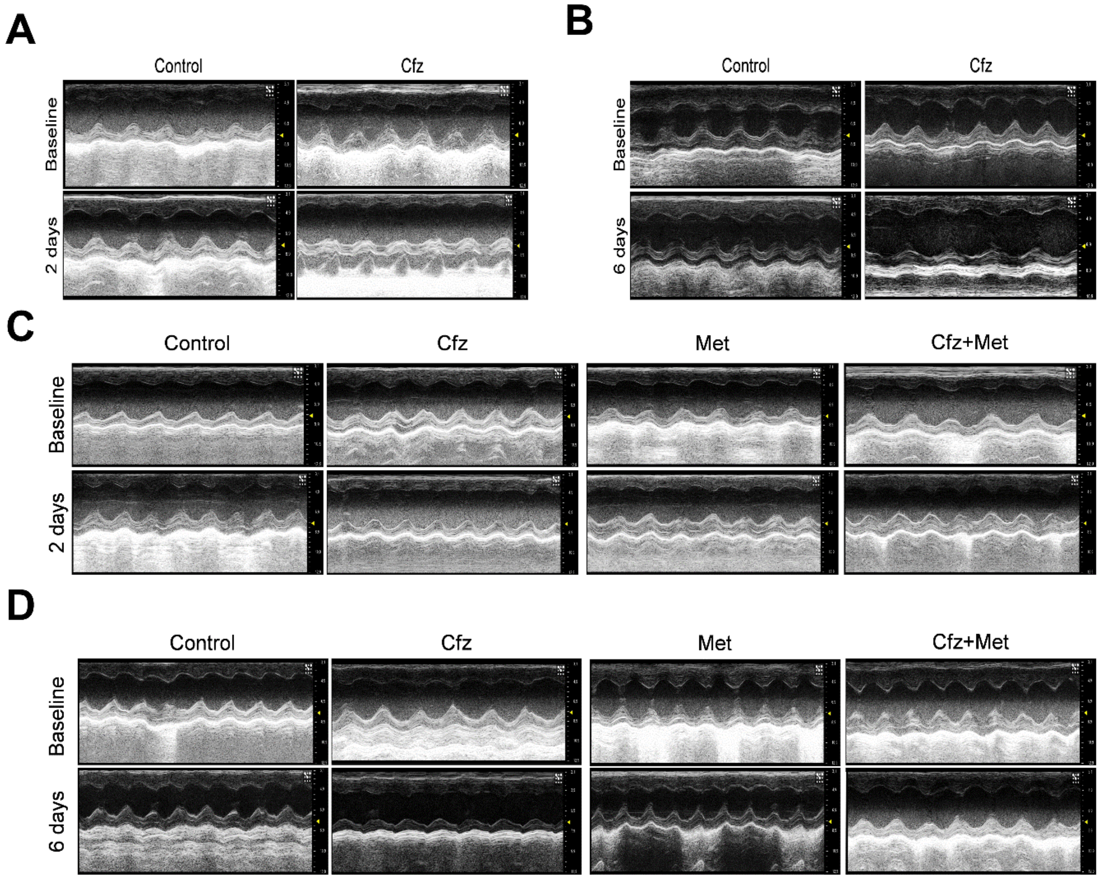

Figure S4: Panel of original echocardiography images. Panel of original echocardiography images including the scale bar corresponding to **A-B.** Figure 1 **C.** Figure S1 and **D.** Figure 4.

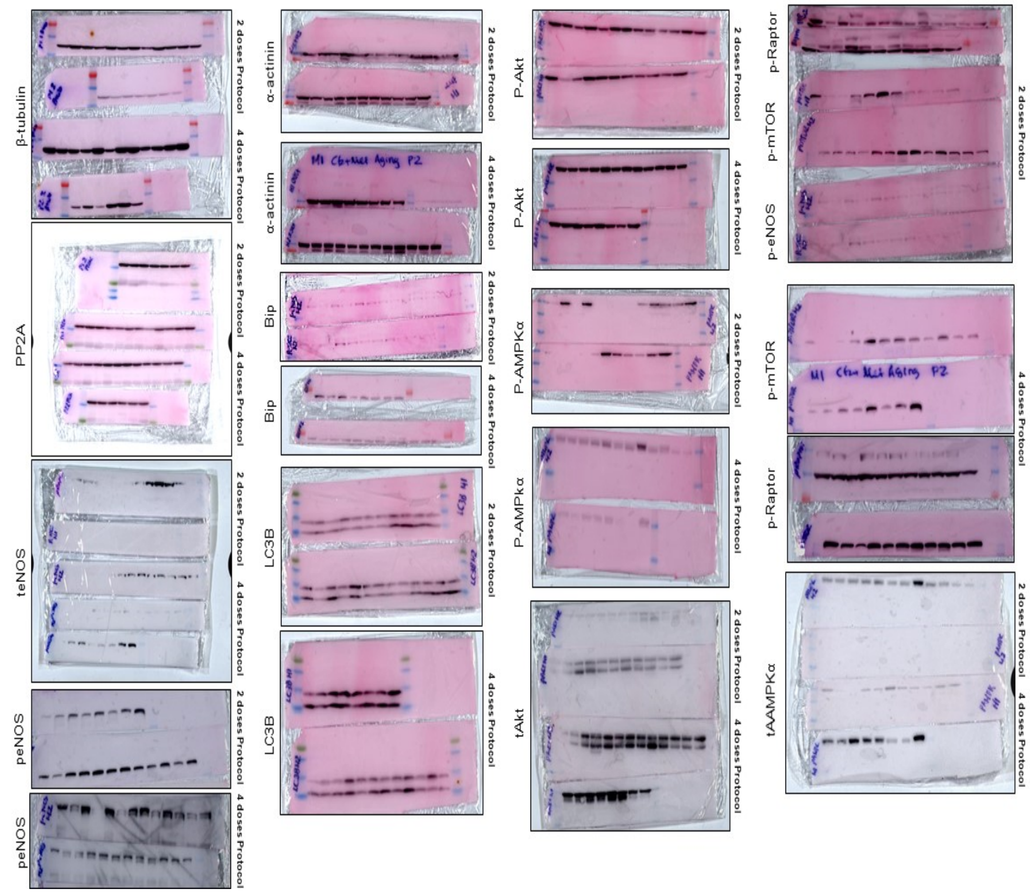

Figure S5: Panel of original western blots. Panel of original western blot images of 2 doses and 4 doses protocols.
